# Tooth Loss Complexity in Partially Edentulous Type 2 Diabetes and Non-diabetic Patients Based on the Prosthodontic Diagnostic Index (PDI) Classification: An Institution-Based Study

**DOI:** 10.7759/cureus.94025

**Published:** 2025-10-07

**Authors:** Joshni Loitongbam, Jogeswar Barman, Komal Sharma, Rajdeep Paul, Barasha Goswami, Ritu Raj Gupta

**Affiliations:** 1 Department of Prosthodontics and Crown &amp; Bridge, Regional Dental College and Hospital, Guwahati, IND

**Keywords:** complexity, non-diabetic, partial edentulism, prosthodontic diagnostic index, tooth loss, type 2 diabetes

## Abstract

Background: The complexity of tooth loss not only compromises oral function and appearance but also has a detrimental impact on oral health-related quality of life. Untreated caries and chronic periodontal disease often lead to partial dentition. This prevalence is further exacerbated by the rising incidence of type 2 diabetes mellitus (DM). These bidirectional effects between diabetes and oral health accelerate tooth loss, necessitating varying levels of prosthodontic intervention to restore oral function and improve patient outcomes. This study aimed to find a correlation between diabetes and the complexity of tooth loss in partially edentulous patients and compare its severity with non-diabetic patients.

Methods: Two hundred partially edentulous patients, non-diabetic (n=100) and diabetic (n=100), reporting to the Department of Prosthodontics, Regional Dental College, Guwahati, India, were classified into four classes according to the Prosthodontic Diagnostic Index (PDI) Classification for partial edentulism. Blood sugar tests were advised as a routine diagnostic procedure.

Results: Diabetic patients showed significantly higher tooth loss complexity (Class III PDI) than non-diabetic participants, as assessed by the PDI classification (p = 0.013). Except for gender, other sociodemographic factors and period of edentulism were all associated with higher tooth loss complexity in the non-diabetic group. PDI Class III was more prevalent in diabetic patients associated with a longer history of diabetes and long-term edentulism. Diabetic urban dwellers demonstrated significantly more complex tooth loss than their rural counterparts.

Conclusion: The complexity of tooth loss was significantly associated with elevated blood glucose level.

## Introduction

Oral health plays a critical role in maintaining general well-being, with tooth loss being one of the significant indicators of poor oral and systemic health, causing functional, esthetic, and social damage. Aggressive periodontitis, non-restorable caries, or any periapical infection are the possible causes of tooth loss and can present adverse consequences on the remaining dentition and on the patients’ general well-being. It is a debilitating and irreversible condition. Venkat et al. gave a systematic review on the prevalence of tooth mortality among adults in India and concluded that the overall prevalence of tooth mortality was 34.6% [1]. Barman et al. (2022) [2] reported that tooth decay is the prime cause of tooth loss (23.7%) in young adults, followed by tooth mobility (4.5%) and trauma (0.9%).

The complexity of losing teeth, especially when it concerns posterior and strategic ones, poses several difficulties. It significantly impairs mastication and speech, especially when anterior teeth are missing. Functionally, it contributes to occlusal instability, causing tooth migration, supra-eruption, and even temporomandibular joint (TMJ) disorders. Collectively, these factors may adversely affect patients' psychological well-being and overall quality of life.

While the causes of tooth loss are multifactorial, diabetes mellitus (DM) is increasingly recognized as a major contributor. According to the International Diabetes Federation Reports (2021) [3], 463 million adults are living with diabetes, and it is estimated to be 578 million by 2030 and 700 million by 2045. Type 2 DM [4] makes up about 90% of all diabetes cases. The prevalence of the disease continues to increase [5], most dramatically in low- and middle-income nations. Numerous complications have been linked to DM in the oral cavity. These include periodontal diseases (periodontitis and gingivitis); salivary dysfunction leading to a reduction in salivary flow and changes in saliva composition; and taste dysfunction. Yu et al. (2024) found a positive correlation between higher HbA1c levels and a greater number of decayed teeth [6]. Other diabetic factors, like reduced blood supply, increase the amount of residual ridge resorption.

Various classification systems have been devised for partial edentulism, with Kennedy's classification being the most common and widely accepted one [7]. But the conditions of the adjacent teeth and supporting structures were not considered in Kennedy's classification [7]. The Prosthodontic Diagnostic Index Classification (PDI), introduced by the American College of Prosthodontists, categorizes partial edentulism from Class I (least complex) to Class IV (most complex) based on diagnostic criteria, aiding in treatment planning [8]. However, research correlating diabetes and tooth loss complexity using PDI is scarce, and no such study has been reported in the Northeast Indian population. Furthermore, little is known about the direct relationship between complexity and sociodemographic factors like age, gender, income, and education, even though these factors are known to affect tooth loss. In order to provide evidence for more focused preventive measures and better prosthodontic planning, this study intends to evaluate the complexity of tooth loss in patients with and without diabetes, as well as its correlation with sociodemographic factors.

## Materials and methods

An institution-based study was conducted in the Department of Prosthodontics and Crown & Bridge of Regional Dental College, Guwahati, Assam, India, affiliated with Srimanta Sankaradeva University of Health Sciences, Guwahati, in collaboration with Pathocare Diagnostic Center, Rupnagar, Guwahati, from August 2023 to February 2025. Two hundred partially edentulous patients reporting to the Department of Prosthodontics, Regional Dental College, Guwahati, seeking prosthodontic rehabilitation were selected for the study according to the inclusion and exclusion criteria. All patients considered for the study were informed in detail about the purpose and study procedure. Written consent was obtained from each subject who voluntarily agreed to participate in the study.

Sample size was determined using the formula: 𝑛=𝑍^2^𝑃⁢(1−𝑃)𝑑^2 ^where n is the sample size, Z is the statistic corresponding to the level of confidence, P is the expected prevalence, and d is precision.

Inclusion criteria

Partially edentulous non-diabetic and type 2 DM patients, irrespective of gender, place of residence, and socioeconomic status, were included in the study.

Exclusion criteria

The study excluded completely edentulous patients, patients with type 1 DM, partially edentulous patients below 18 years of age, partially edentulous patients recently diagnosed with type 2 DM, non-ambulatory patients, and those with congenital and acquired maxillofacial defects and a history of fracture and tooth loss due to therapeutic treatment like orthodontic and prophylactic extraction of wisdom tooth/teeth.

Study design

This institution-based study was conducted based on the steps outlined in Table 1.

**Table 1:** Study design

Step	Description
1	Collection of information using a structured proforma consisting of sociodemographic factors, such as age, gender, socioeconomic status, and demographic background.
2	Collection of clinical data from the participant’s medical and dental histories.
3	Intraoral examination was conducted under the cross-infection control protocols for each subject.
4	Screening and classification of subjects into four classes according to the Prosthodontic Diagnostic Index Classification (PDI) criteria for partial edentulism based on complete history, clinical and radiographic examination, and systematic evaluation of each patient.
5	Monitoring of blood glucose level as a routine diagnostic procedure by advising Hb1Ac for both test groups and glucose tolerance test for the non-diabetic, while fasting and post-prandial glucose test for the diabetic subjects.
6	Explanation to patients regarding the blood sample collection procedure, with instructions to report to the laboratory in a fasting state
7	An orthopantomogram (OPG) was advised to determine the periodontal status of the remaining teeth and the quantity of residual alveolar bone.

Data collection

Information on the socio-demographic and clinical variables was collected during history taking and clinical examination, and coded accordingly following the WHO’s oral health assessment [9] format, 1997. Laboratory data for blood sugar level, such as HbA1c, glucose tolerance test (GTT), and fasting and post-prandial tests, were collected for each of the participants. Self-reported non-diabetic participants showing an HbA1c value of >6.5% and a two-hour plasma glucose level ≥200 mg/dL were excluded from the study following the exclusion criteria. The data were summarized using frequency distribution tables and cross-tabulations, showing relative percentages. The frequency distributions were tested for statistical uniformity using the chi-square test for goodness of fit at the 0.05 significance level. The cross-tables showing the interaction of two study variables were tested for association using Pearson's chi-square test at the 0.05 significance level. In all tests, a p-value less than 0.05 is considered statistically significant. All the collected data were entered into Microsoft Office Excel 2007 version (Microsoft Corporation, Redmond, WA) and subsequently analyzed using IBM SPSS Statistics software for Windows, version 20.0 (IBM Corp., Armonk, NY).

## Results

The total sample population was 59.5% males and 40.5% females, representing all religions, castes, and creeds. Chi-square analysis was done using IBM SPSS Statistics software. All the tests were conducted at the 0.05 significance level. The inferences are drawn with the help of the p-value.

Tables 2, 3 illustrate the frequency distribution of partial edentulism among diabetic and non-diabetic participants based on the PDI classification. In the overall study population, the distribution of tooth loss complexity within the non-diabetic group was not statistically significant (p = 0.971). However, the diabetic group demonstrated a highly significant association (p < 0.001), with PDI Class III being the most prevalent classification.

**Table 2:** Frequency distribution of tooth loss complexity among non-diabetic, partially edentulous patients according to the PDI classification *chi-square test value = 0.240; df = 3, p=0.971 (statistically non-significant) PDI: Prosthodontic Diagnostic Index Classification

Non-diabetic group	PDI classification	Frequency	P-value*
	1	26	0.971
	2	25	
	3	26	
	4	23	
	Total	100	

**Table 3:** Frequency distribution of tooth loss complexity among partially edentulous type 2 diabetic patients according to the PDI classification *chi-square test value = 23.20; df = 3, p<0.001 (statistically significant) PDI: Prosthodontic Diagnostic Index Classification

Diabetic group	PDI classification	Frequency	P-value*
	1	18	P <0.001
	2	12	
	3	44	
	4	26	
	Total	100	

Table 4 depicts the complexity of tooth loss among type 2 DM and non-diabetic partially edentulous patients with respect to PDI classification. The findings indicate that diabetic patients exhibited a higher complexity of tooth loss compared to non-diabetic individuals, particularly Class III (62.9%) and Class IV (53.1%). Pearson’s chi-square test revealed a statistically significant difference in the prevalence of tooth loss complexity between the two study groups (p = 0.013).

**Table 4:** Complexity of tooth loss among type 2 diabetic and non-diabetic partially edentulous patients according to the PDI classification * Pearson’s chi-square test value = 10.834; df =3, p = 0.013 (statistically significant) PDI: Prosthodontic Diagnostic Index Classification

		PDI classification				Total	P-value*
		I	II	III	IV		
Non-diabetic patients	Count	26	25	26	23	100	0.013
	%	59.1%	67.6%	37.1%	46.9%	50.0%	
Diabetic patients	Count	18	12	44	26	100	
	%	40.9%	32.4%	62.9%	53.1%	50.0%	
Total	Count	44	37	70	49	200	
	%	100.0%	100.0%	100.0%	100.0%	100.0%	

The results of the present study revealed a statistically significant difference in tooth loss complexity between type 2 DM and non-diabetic patients, with a p-value of 0.013, with a higher prevalence of PDI Class III.

In this study, severe tooth loss was significantly associated with older age (≥60 years), while gender showed no notable influence. Among the diabetic patients, urban residents had more Class III and IV patterns, possibly due to healthcare access or lifestyle differences. Lower socioeconomic status was linked to a higher prevalence of Class IV tooth loss.

## Discussion

Tooth loss is a serious public oral health concern, with its complexity varying among individuals [10] and impacting aesthetic, functional, psychological, and social well-being. Dental caries is the most common cause of tooth loss in adults, followed by periodontal disease, where the formation of a biofilm on the tooth surface is a major etiological factor [11]. If untreated, gingivitis can progress to periodontitis and ultimately to tooth loss. Other contributing factors include age, smoking, and systemic conditions such as DM.

Edentulism results in a number of oral complications, including drifting, supraeruption, facial asymmetry, and collapse of the oral structures, making prosthodontic rehabilitation complicated due to continued loss of alveolar bone and supporting tissues. The number and location of missing teeth, sociodemographics, and treatment delays, primarily for patients with their anterior teeth intact, all have an impact on perceptions of need and thus influence patient management.

Glycemic control strongly influences oral health, as high blood sugar promotes bacterial growth, infections, and inflammation, leading to tissue destruction and tooth loss. Individuals with ≥6 mm periodontal pockets are 3.5 times more likely to develop type 2 DM than those with shallower pockets [12, 13].

This study assessed partial edentulism complexity in type 2 DM and non-diabetic patients using the PDI, examining links with sociodemographics, edentulism duration, and diabetic history, highlighting its multifactorial nature and association with systemic conditions like DM.

Consistent with previous literature, this study found more severe tooth loss in diabetics, with a higher prevalence of PDI Class III. Luo et al. (2015) [14] reported that diabetic adults had nearly twice the number of missing teeth compared to non-diabetics, with tooth loss disparities persisting over time and being most pronounced in non-Hispanic Black populations. Kapp et al. (2007) [15] examined that respondents with diabetes were 1.46 times more likely (95% CI, 1.30-1.64) to have at least one tooth removed than respondents without diabetes. Similarly, Suzuki et al. (2020) [16] and Hawsawy et al. (2019) [17] observed greater molar loss and higher prevalence of Kennedy Class I patterns in diabetics, particularly in the mandibular arch.

Glycemic control plays a crucial role in the severity of periodontal disease and subsequent tooth loss. Poorly controlled diabetes (HbA1c > 8%) has been associated with a greater number of missing teeth compared to well-controlled diabetes (HbA1c < 7%) [18]. Systemic diseases with increased inflammation are frequently linked to increased risk of periodontal disease. In fact, the American Dental Association reported that one in five cases of tooth loss is related to diabetes [19]. This present study aligned with these findings, as Class III and IV PDI categories were notably more frequent in diabetic patients, likely due to the unrecognized bidirectional relationship between diabetes and periodontal disease, where hyperglycemia worsens periodontitis, and periodontitis increases insulin resistance, accelerating tooth loss. indicating greater complexity in tooth loss and severity in prosthodontic rehabilitation.

Gender differences were not statistically significant in this study, consistent with Jeyapalan et al. (2015) [20]. However, Khan et al. (2019) [21] found males had more missing teeth, while others noted women were more likely to seek restoration due to greater aesthetic awareness and had better health-seeking behavior.

In this study, non-diabetic rural residents showed more complex tooth loss (Class III PDI), while among diabetics, urban dwellers had more Class III and IV patterns, possibly due to differences in healthcare access or lifestyle. In India, there is a gross disparity in oral health care provision between urban and rural areas [22]. Availability, accessibility, and affordability of dental services might be the potential barriers for rural people to seek timely advice and treatment, and ethnic and regional variations influencing tooth loss complexity as described by Luo et al. (2015)[14], Weijdijk et al. (2020)[23], and Deguchi et al. (2017)[24].

In non-diabetic patients, lower SES, based on combined employment and income, was linked to a higher prevalence of complex tooth loss (Class IV PDI), consistent with the conclusion by Vadavadaga (2015) [25] that financial constraints delay care and increase disease burden. There is a definitive need for a step-by-step approach in eradicating the cause all over the country, with special focus on people who suffer from socio-economic and geographical disadvantage. In diabetics, socioeconomic status showed no significant association, likely due to uniformly high tooth loss risk, though education level significantly influenced awareness of tooth loss consequences.

In diabetics, longer durations of diabetes (>10 years) or edentulism (>3 years) were associated with a higher prevalence of Class III and IV PDI patterns, suggesting cumulative effects of chronic hyperglycemia, though statistical significance was lacking. Literature indicates that tooth loss, rather than diabetic status, more strongly impacts oral health-related quality of life, emphasizing the importance of timely prosthetic rehabilitation. The PDI classification effectively assessed prosthodontic needs, with diabetics showing greater complexity. Chi-square analysis revealed a significant difference in tooth loss complexity between diabetics and non-diabetics (p = 0.013). Study strengths include systematic PDI-based classification and correlation of tooth loss complexity with sociodemographic factors and duration of edentulism.

However, limitations of this study include a relatively modest sample size, unequal variable distribution, and exclusion of other compounding factors like history of smoking, oral hygiene practices, dietary habits, and systemic comorbidities other than DM, which may affect the rate and complexity of tooth loss. Therefore, to strengthen the evidence and validate these findings, larger longitudinal cohort studies may be recommended to better assess the correlation between tooth loss complexity, diabetes, and sociodemographic determinants.

## Conclusions

Within the limitations of the study, type 2 DM patients exhibited significantly greater tooth loss complexity, particularly in PDI Classes III and IV, compared to their non-diabetic counterparts. However, gender differences in both diabetic and non-diabetic groups were not statistically significant, indicating a similar pattern of tooth loss complexity amongst male and female participants. Socioeconomic status showed a statistically significant association with tooth loss complexity in non-diabetic patients, suggesting that individuals from lower socioeconomic strata were more likely to exhibit higher PDI classes. Among diabetics, urban residents showed significantly more complex partial edentulism than rural residents.
